# Subacute Cutaneous Lupus Erythematosus-Like Eruption Induced by EGFR -Tyrosine Kinase Inhibitor in *EGFR*-Mutated Non-small Cell Lung Cancer: A Case Report

**DOI:** 10.3389/fmed.2021.570921

**Published:** 2021-06-04

**Authors:** Alessandra Ferro, Angela Filoni, Alberto Pavan, Giulia Pasello, Valentina Guarneri, PierFranco Conte, Mauro Alaibac, Laura Bonanno

**Affiliations:** ^1^Department of Surgery, Oncology and Gastroenterology, Università degli Studi di Padova, Padova, Italy; ^2^Medical Oncology 2, Istituto Oncologico Veneto Istituto di Ricovero e Cura a Carattere Scientifico (IRCCS), Padova, Italy; ^3^Melanoma and Sarcoma Surgical Oncology Unit - Veneto Institute of Oncology, IOV-Istituto di Ricovero e Cura a Carattere Scientifico (IRCCS), Padua, Italy; ^4^Unit of Dermatology, University of Padua, Padua, Italy

**Keywords:** osimertinib, immunotherapy, targeted therapy, cutaneous drug reactions, immune- related adverse events

## Abstract

EGFR tyrosine kinase inhibitors (TKIs) are the front-line treatment in *EGFR* mutation positive advanced non-small cell lung cancer (aNSCLC) patients. Generally, they are well-tolerated but skin toxicity is common (45–100% of patients) and may adversely affect quality of life. Pathogenesis of cutaneous side effects is usually linked to EGFR expression in normal cells of the epidermis and not immune-related. Subacute cutaneous lupus erythematosus (SCLE) is an autoimmune disease and about 40% of SCLE cases are drug related, but no reports are available involving osimertinib. Our report depicts a drug induced-SCLE (DI-SCLE) caused by erlotinib and worsened by osimertinib. The adverse event is characterized by the absence of systemic symptoms. Diagnosis has been performed by skin biopsy and the conditions improved with systemic steroids administration and EGFR-TKIs discontinuation. The report underlines the importance of a complete dermatologic diagnosis of skin lesions induced by EGFR inhibitors, according to symptom severity and timing of improving with standard clinical management. The diagnosis of immune-related skin toxicity in this context affects the treatment and the outcome of skin toxicity and must be taken into account when planning subsequent treatments, potentially including immune checkpoint inhibitors (ICIs).

## Introduction

Epidermal growth factor receptor tyrosine kinase inhibitors (EGFR TKIs) are currently the standard of care for front-line treatment in advanced non-small cell lung cancer (NSCLC) patients carrying *EGFR* sensitizing mutations. Both first and second generation TKIs demonstrated to be superior to chemotherapy in terms of response rate (RR) and progression-free survival (PFS) (1–8). A third-generation irreversible EGFR TKI, osimertinib, has been developed to overcome acquired resistance to EGFR TKI driven by *EGFR* T790M mutation (9). Subsequently, because of its superiority to first generation EGFR TKIs in terms of RR, PFS and overall survival (OS), independently from the presence of T790M mutation, it has been recently approved as first-line treatment for advanced *EGFR*-mutated NSCLC (10, 11).

The most common drug-related side effects of EGFR TKIs are acneiform skin rash, follicular papulopustular eruption, xerosis, diarrhea, and paronychia (12). Cutaneous toxicity is usually a dose-dependent skin drug reaction, which develops after 1–2 weeks of treatment, peaks at 3–4 weeks on therapy, and its intensity decreases after 2 weeks but can often persist over some months (13). Despite lower systemic toxicity than conventional chemotherapy, the majority of patients treated with these agents experiences cutaneous disorders, because of the ubiquitous presence of EGFR in the skin and its involvement in maintaining the integrity of the tissue. Specifically, EGFR is expressed in undifferentiated, proliferating keratinocytes in the basal and suprabasal layers of the epidermis. The inhibition of EGFR catalytic activity induces growth arrest and apoptosis, decreased cell migration, increased cell attachment and differentiation and also stimulates inflammation, resulting in typical cutaneous lesions (14).

Subacute cutaneous lupus erythematosus (SCLE) is a subtype of cutaneous lupus erythematosus that was first described in 1979 by Sontheimer et al. (15). Skin lesions typically manifest as papulosquamous or annular eruptions developing in sun-exposed areas. SCLE usually occurs in genetically predisposed individuals, primarily in young to middle aged women, frequently associated with the presence of the anti-Ro (SS-A) autoantibody, a positive antinuclear antibody (ANA) reaction and human leukocyte antigens B8, DR3, DRw52, and DQ1 (16).

About 40% of SCLE cases may be drug related (DI-SCLE) (17). Proton pump inhibitors, antifungal medications, hydrochlorothiazide and calcium channel blockers are the most common drugs involved, but some chemotherapeutic agents have been more recently implicated (18, 19). To the best of our knowledge, in literature there is only one other case report of SCLE-like eruption induced by an EGFR TKI (erlotinib) and no one linked to osimertinib (20). Moreover, clinical manifestation of this immune-related toxicity is different from the previously reported one and has important implications in clinical management.

## Case Report

In July 2018, a 71-year-old woman with no smoking habit and few comorbidities (arterial hypertension and autoimmune hypothyroidism) was diagnosed with stage IV lung adenocarcinoma with metastases to lung, liver, bone, adrenal glands and lymph nodes (T4N3M1c according to 8th edition of TNM) (21). A tissue-based mutation testing by using Real-time PCR (Easy EGFR, Diatech Pharmacogenetics) showed an *EGFR* mutation in exon 21 (L858R point mutation).

**Figure 1:**
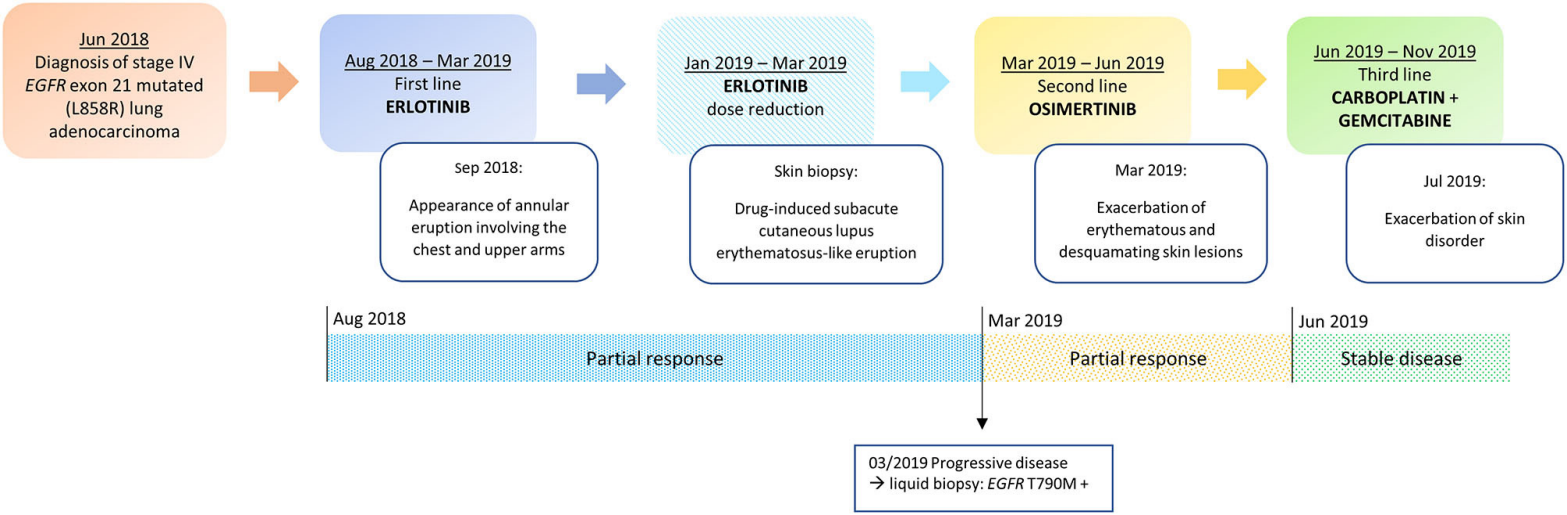
Timeline of disease status, corresponding treatments administered and development of skin toxicity.

A targeted therapy with erlotinib 150 mg daily was started and since the second cycle the patient experienced severe pruritus and developed a grade 2 cutaneous adverse event (AE), characterized by the appearance of an annular eruption involving the chest and upper arms (Figure 1). The toxicity was managed with the administration of topical corticosteroids (betamethasone dipropionate 0.05% ointment) and oral antihistamine (cetirizine 10 mg/daily) with partial clinical benefit. After three cycles the radiological evaluation with computerized tomography (CT) scan showed a partial response according to the Response evaluation criteria in solid tumors (RECIST) v1.1 (22).

**Figure 2:**
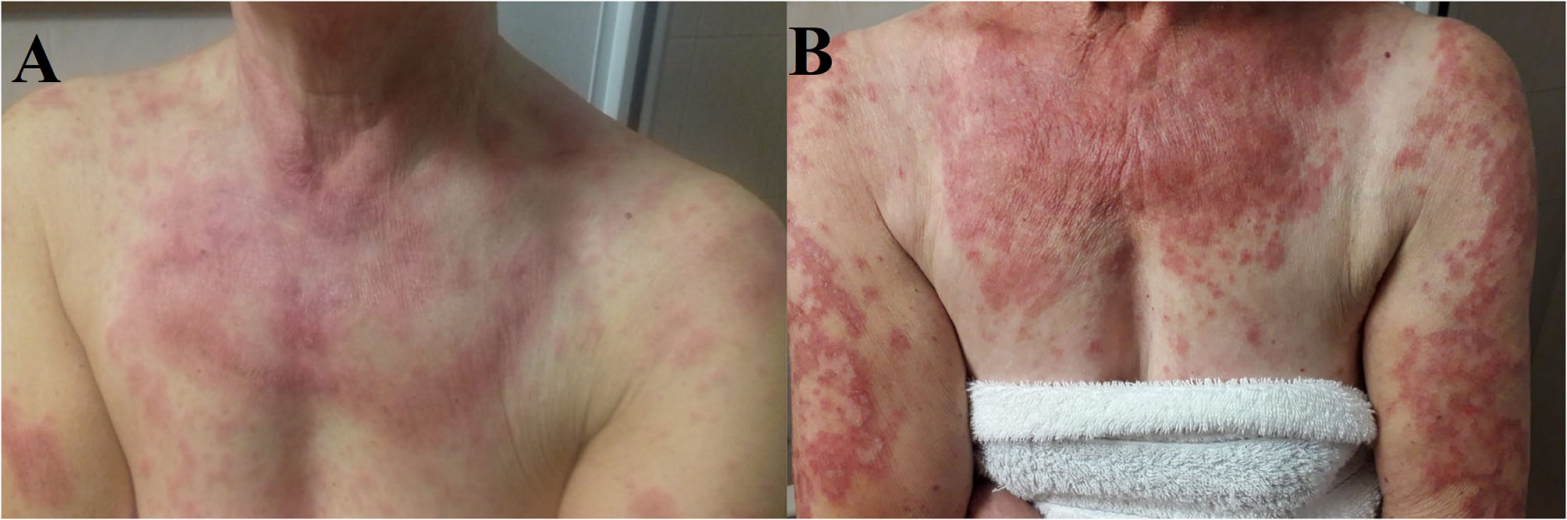
**(A)** Cutaneous eruption composed of annular, palpable, pink, scaly plaques on the patient's upper chest, and arms. **(B)** Numerous erythematous annular plaques on the chest and arms with a typically photo distributed pattern.

In November 2018, because of the persistence of the cutaneous toxicity, the patient underwent first dermatologic consultation: the skin lesions were considered as related to erlotinib and topical corticosteroids were administered. However, the skin disorder worsened, evolving to a grade 3 maculopapular rash, seriously affecting her quality of life. Therefore, erlotinib was temporarily discontinued and new dermatologic evaluation was planned after 1 week. Physical examination revealed numerous annular lesions, with raised pink borders and central clearing on the chest and upper arms (Figure 2A). After treatment interruption and topic treatment, the rash improved and the treatment was resumed.

In January 2019, skin lesions worsened again and erlotinib was prescribed at reduced dose of 100 mg daily. In addition, given clinical suspicion of DI-SCLE, in January 2019 a biopsy of a right supraclavicular cutaneous lesion was performed. The pathology report revealed the presence of interface dermatitis with parakeratosis and focally thinning of the epidermis and a diffuse dyskeratosis. A perivascular and interstitial lymphocytic infiltrate and vacuolar degeneration of the basal layer was described in the dermis. Furthermore, laboratory findings revealed speckled-pattern antinuclear antibodies (ANA) at 1: 160 (normal >1: 40) and anti-endomysial antibody (EMA) positivity. This set of morphological features together with the clinical-anamnestic characteristics suggested a diagnosis of DI-SCLE-like eruption. For this reason, the patient was prescribed a course of systemic corticosteroids (prednisone 25 mg/daily) and antihistamine in combination with topical steroids. This treatment was effective, providing a prompt reduction of symptoms and signs, allowing for progressive steroid tapering and discontinuation.

In March 2019, after 10 cycles of erlotinib treatment, the CT scan showed disease progression. According to guidelines, a liquid biopsy was thus performed and *EGFR* T790M acquired resistance mutation was detected in circulating tumor DNA by using Real-time PCR (Cobas cfDNA Roche). Based on this result, second line treatment with the third generation TKI osimertinib was initiated (Figure 1).

Since the first cycle, the patient underwent a dramatic exacerbation of the itchy, erythematous and desquamating skin lesions, which involved the face and the chest (Figure 2B). Osimertinib was the only identifiable precipitant of the cutaneous AE. The dermatologist prescribed a systemic therapy with prednisone 25 mg per day, antihistamine and a short-course of antibiotic therapy with minocycline 100 mg bid in association with topical steroid treatment. Again, the prompt relief of the clinical manifestation led to steroid tapering and antihistamine interruption.

In June 2019 the first radiological revaluation showed a progressive disease, so a third line therapy with carboplatin and gemcitabine was started. After one cycle of chemotherapy the patient experienced a recurrence of skin lesions and she underwent a new dermatological evaluation. Systemic therapy with prednisone 25 mg per day for 2 weeks was prescribed and skin lesions progressively improved (Figure 1).

After five cycles of therapy the patient underwent a sudden clinical worsening and extensive liver disease progression, resulting in decline of patient's general condition and death.

In April 2019 the patient provided written informed consent to the publication of her anonymous case report including photos and details about her disease.

## Discussion and Conclusions

Since DI-SCLE was first described, several drugs have been identified as involved with its development. Among oncological treatment, some chemotherapeutic agents, such as taxanes, pyrimidine analogs, gemcitabine, and anthracyclines have been identified as sources/causative agents of SCLE (23, 24). More recently also some cases of lupus erythematosus-like eruption induced by targeted therapies have been reported: one related to the multikinase inhibitor pazopanib, another one related to the cyclin-dependent kinases 4 and 6 inhibitor palbociclib and two associated with the anti-vascular endothelial growth factor (VEGF) monoclonal antibody bevacizumab (25, 26).

In the era of personalized medicine, the use of targeted therapies has led to a therapeutic revolution in particular for molecularly defined subsets of NSCLC. Despite lower systemic side effects than chemotherapy, TKIs may cause different cutaneous AEs including dry skin, dermatitis acneiform, folliculitis, paronychia, alopecia, pruritus, and xerosis. The discomfort caused by these AEs can reduce compliance to anti-EGFR therapy and therefore affect patients' outcome. For this reason, prompt and proper management of skin toxicity is essential in clinical practice. International guidelines do not consider skin lesions biopsy as necessary for toxicity management (27).

DI-SCLE is an autoimmune disease that manifests especially on the upper body including face, neck and trunk (sun-exposed areas) with annular or papulosquamous presentation with histologic findings of interface dermatitis with focal vacuolization of the epidermal basal layer associated with a perivascular dermal lymphocytic infiltrate. The pathogenic mechanisms involved in DI-SCLE remains unclear: several hypotheses have been proposed, including genetic predisposition, drug biotransformation, and epigenetic dysregulation in immune cells (28).

**Figure 3:**
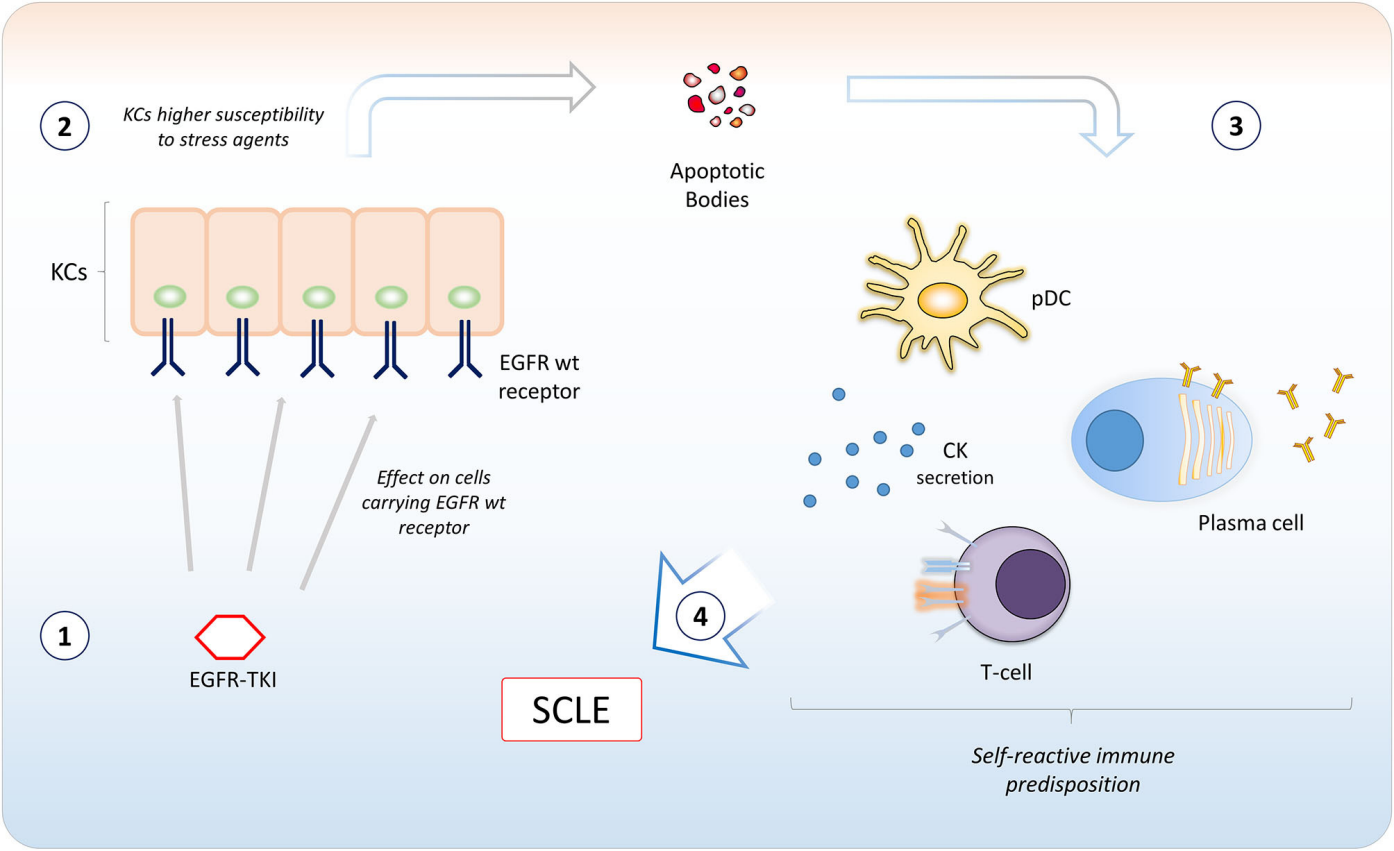
A model for potential involvement of epidermal growth factor receptor tyrosine kinase inhibitors (EGFR TKIs) in the pathogenesis of Subacute Cutaneous Lupus Erythematosus. EGFR TKIs bind EGFR expressed in keratinocytes (KCs) in the basal and suprabasal layers of the epidermis (1). The inhibition of EGFR catalytic activity induces apoptosis or could sensitize KCs to agents inducing cellular stressing conditions (2). This causes the ignition of an inflammatory microenvironment, with recruitment of different immune cells. A self-reactive predisposition of the immune system (e.g., certain MHC polymorphisms) (3) may confer susceptibility to subacute cutaneous lupus erythematosus (SCLE) development (4). CK, cytokines; pDC, plasmacytoid dendritic cell.

In our case, the SCLE-like lesions were likely to be induced by osimertinib therapy. Even though the mechanism is not known, a potential involvement of EGFR TKIs in the pathogenesis of SCLE might be related to the inflammation induced by apoptosis and cellular stressing conditions following EGFR inhibition in subjects with self-reactive predisposition of the immune system (Figure 3).

Although our patient did not perform serological dosage of Anti-Ro/SSa antibodies, the clinical picture and skin biopsy was consistent with DI-SCLE-like eruption and the most likely cause of the exacerbation of skin lesions was the administration of osimertinib after erlotinib, on the basis of the close temporal relationship between drug exposure and symptoms' onset.

To our knowledge, only another case of SCLE -like eruption related to anti-EGFR therapy has been reported: Takeda et al. described the case of a patient diagnosed with *EGFR*-mutated NSCLC who received erlotinib (150 mg daily) as a third-line therapy (20). After 2 weeks of treatment, she manifested fever, multiple erythematous patches over her upper chest and upper limbs, and prominent butterfly-shaped plaque erythema over her malar eminences that was categorized as lupus erythematosus-like eruption (Grade 3). A skin biopsy specimen from the upper chest revealed superficial perivascular dermatitis with a vacuolar change consistent with cutaneous lupus erythematosus. Also in this patient anti-Ro/SSa antibodies were not examined.

Our case suggests novel issues. The patient had no systemic symptoms and no butterfly-shaped erythema, thus underlining the importance of considering skin lesions biopsy in case of persistent skin toxicity, after dermatologic evaluation and multidisciplinary discussion. In addition, the toxicity worsened with the administration of osimertinib, usually associated with more favorable skin toxicity profile when compared to first- and second-generation EGFR TKIs.

The potential involvement of osimertinib in the pathogenesis of an autoimmune disorder (Figure 3) is particularly relevant. Indeed, nowadays osimertinib represents a standard of care in first-line setting for advanced *EGFR*-mutated NSCLC (10, 11). Moreover, *EGFR*-mutated patients might be also considered for a treatment with immune checkpoint inhibitors (ICIs) following EGFR TKI. Even though *EGFR* mutations are generally associated with less effectiveness of ICIs, these drugs might be considered after EGFR TKIs and at least one chemotherapy treatment (29, 30). Furthermore, atezolizumab in combination with bevacizumab, paclitaxel and carboplatin might also have a role for patients progressing to first or second line osimertinib (31).

It is well-known that the blockade of regulatory immune checkpoint can result in aberrant immune activation leading to undesirable inflammation and autoimmunity (32). Recently, SCLE has been reported to occur during anti-PD-1 and anti-PD-L1 immunotherapy (33–35). In this context, the diagnosis of previous immune-related toxicity associated with EGFR TKI should be taken into account in decision making for systemic treatment in patients progressing to osimertinib and caution is needed in order to avoid serious immune-related AEs during treatment with ICIs.

In conclusion, we describe immune-related skin toxicity induced by erlotinib and worsened by osimertinib. The observation suggests the importance of multidisciplinary evaluation in case of persistent moderate-severe skin toxicity. Considering skin biopsy and potential immune-related pathogenesis is important not only for the management of skin toxicity and subsequently the compliance to EGFR TKI treatment, but also in planning further lines of treatment, potentially including immunotherapy.

## Data Availability Statement

The raw data supporting the conclusions of this article will be made available by the authors, without undue reservation.

## Ethics Statement

The patient provided written informed consent to the publication of her anonymous case report including photos and details about her disease.

## Author Contributions

AFe and LB conceived the manuscript. AFe, AP, and LB collected clinical, radiological data, and wrote the manuscript. LB and GP were responsible for the patient' care. AFi and MA were the dermatologists in charge of the patient, provided imaging, and critical review. VG and PC performed editing and critical review. All the authors read and approved the manuscript.

## Conflict of Interest

The authors declare that the research was conducted in the absence of any commercial or financial relationships that could be construed as a potential conflict of interest.
